# Applicability of Flours from Pigmented and Glutinous Rice in Gluten-Free Bread Baking

**DOI:** 10.3390/foods12061324

**Published:** 2023-03-20

**Authors:** Iva Burešová, Libor Červenka, Romana Šebestíková, Monika Augustová, Alžbeta Jarošová

**Affiliations:** 1 Faculty of Technology, Tomas Bata University in Zlín, Nám. T. G. Masaryka 5555, 760 01 Zlín, Czech Republic; 2 Faculty of Chemical Technology, University of Pardubice, Studentská 95, 532 10 Pardubice, Czech Republic; 3 Faculty of AgriSciences, Mendel University in Brno, Zemědělská 1, 613 00 Brno, Czech Republic

**Keywords:** black rice, red rice, glutinous rice, dough, slurry, rheology, bread quality, texture, applicability

## Abstract

The flours from pigmented and glutinous rice have a great potential to increase the nutritional quality of gluten-free breads. The characteristics of whole-meal fine flours, slurries, doughs, and breads prepared from black, red, and white glutinous rice were, therefore, compared with commercially used refined fine and semi-coarse white rice flours. The pasting properties of different flours were strongly influenced by the type of rice they were made from. Slurries with red and glutinous flours exhibited a shift to a lower pasting temperature *T*_0_, lower values of *η_Peak_* and *η_Final_*, as well as higher values of the breakdown and setback region than the slurries with refined flours. The slurry with black flour exhibited high values of viscosity *η*_0_ and breakdown, together with low values of *η_Final_*, setback region and total setback. Bread characteristics were strongly correlated with the pasting properties. The presence of bran particles did not negatively impact loaf volume, crumb hardness, springiness, and chewiness. Some defects were observed in glutinous and red rice bread. Fine flour exhibited better baking performance than semi-coarse flour. Glutinous flour has the potential to become an ingredient in gluten-free baking. The applicability of various black and red rice flours may be limited by the flavor and the taste.

## 1. Introduction

Research into gluten-free bread was previously focused mainly on increasing dough’s ability to trap leavening gas, increasing bread volume, and preparing bread with a softer crumb. Commercially baked breads have low nutritional value as they are mainly prepared from starch blends. This is a significant problem that requires solving since a strict gluten-free diet is often necessary throughout a person’s entire life. Our research is, therefore, focused on the applicability of nutritionally valuable rice grains in gluten-free baking.

Rice (*Oryza sativa* L.) flour is used for cakes, puddings, and noodles. It may also replace wheat flour in bread making. Flour that is prepared from white rice exhibits several significant properties, such as bland taste, colorlessness, and hypoallergenic characteristics, which make it suitable for gluten-free bread making. Low levels of protein, sodium, fat, fiber and a high amount of easily digestible carbohydrates were also reported [1,2].

Anthocyanin pigments situated in the grain coat impact the color of rice grains (black and red) [3]. Pigmented rice grains have a higher phenolic content than white rice grains. Since the phenolic content is positively correlated with antioxidant activity, the antioxidant properties are also higher [4,5]. Pigmented rice is a rich source of vitamin E; red-colored grains are rich in iron and zinc; black varieties are high in protein, fat and crude fiber [3,6]. The phenolic constituents are not homogeneously distributed. Higher levels can be found in the brans and embryo than in the endosperm [7]. Therefore, these parts of grain have to pass into the flour to obtain whole-meal flour, which may be useful in making various products with health benefits [5]. The presence of bran particles in flours may negatively influence bread volume and crumb hardness [8,9]. The flours from pigmented rice also have a great potential to increase the nutritional quality of gluten-free food, which is generally low in fibers, proteins, micronutrients, vitamins, and minerals such as iron, zinc, magnesium, and calcium [10]. The significant differences in the chemical composition (moisture, ash, crude fat, protein, fiber, and carbohydrate content), physicochemical, rheological, and functional properties reported by Tangsrianugul et al. and Kraithong et al. [11,12] may limit the applicability of these rice types in food production.

White glutinous rice (*Oryza sativa* var. glutinosa) is a type of rice that is mainly cultivated in Asia [13]. This rice is white, sticky, and opaque after cooking [14]. The very low content of amylose influences the starch gelatinization temperature, pasting consistency, and extent of retrogradation [15], which may significantly impact the bread texture. The high amounts of protein, dietary fiber, essential amino acids, and essential fatty acids [16,17] make this flour suitable for increasing the nutritional value of gluten-free bread.

The rice grain consists of the hull, bran, endosperm, and embryo. The bran layer is rich in dietary fiber, minerals, and vitamin B complex. The endosperm contains starch and proteins. The objective of white rice milling is to remove hull, bran, and embryo. The remaining endosperm is ground to obtain refined flour of desired granulation [6,18]. The flour granulation may influence bread quality, mainly bread volume [19,20]. The milling results in the loss of some nutritionally valuable substances. Moreover, the anthocyanin pigments are situated mainly in the grain coat (bran). The rice milling procedure may, therefore, be modified to remove only the hull. The remaining parts of grains are milled together to obtain nutritionally valuable whole-meal rice flour.

Besides the nutritional value and granulation, the performance of flour during bread baking is a key factor influencing the applicability of flour in gluten-free bread production. Therefore, the goal of this study was to assess the applicability of the flours prepared from black, red, and glutinous rice in gluten-free bread making. The characteristics of flours, slurries, doughs, and breads were compared with slurries, doughs and breads prepared from commercially used refined white rice flours. The impact of flour granulation was also evaluated. The types of rice involved in this study were selected according to the previously published grain characteristics, as well as rice availability in the Czech market.

## 2. Materials and Methods

### 2.1. Flours

Black, red, white glutinous and common white rice grains were bought from a local supplier. Each type of rice was in the form of a commercial blend. Information about the rice varieties used in the blends was not available. The objective of the black, red, and glutinous rice milling was to remove the hull; de-hulled grains containing bran and endosperm were ground using a pin mill (FF Servis spol. s r.o., Prague, Czech Republic). The ground material was sieved using the AS200 Control (O.K. servis BioPro, Prague, Czech Republic) machine to obtain fine flours. The objective of the white rice milling was to remove hull, bran, and embryo. The remaining endosperm was ground using a pin mill (FF Servis spol. s r.o., Prague, Czech Republic), and the ground material was sieved to obtain refined white fine and semi-coarse flours. The flours were vacuum-packed in polyethylene bags and stored at 4 °C before further analysis was conducted.

All fine flours were characterized by being made up of at least 96% of particulate that passed through a 257 μm sieve opening and a maximum of 75% of particulate that passed through a 162 μm sieve opening. Semi-coarse flour requires at least 96% of particulate to pass through a 366 µm sieve opening and a maximum of 75% of particulate to pass through a 162 μm sieve opening.

### 2.2. Flour Characteristics

The contents of starch and proteins in flour were determined according to ISO 10520 [21] and ISO 1871 [22]. The activity of amylase enzymes in the flours was expressed as Hagberg falling number according to ISO 3093 [23]. At least six measurements were performed on each of the tested flours. The content of sugars (reducing and nonreducing) in the flour was determined according to AOAC Official Method 939.03 [24] and expressed as the content of maltose. The content of damaged starch was determined by a starch damage assay kit (Megazyme, Ireland).

The microstructure of the flour samples was evaluated using a scanning electron microscope (Vega3 SBU, Tescan, Brno, Czech Republic). Gold-plated flour samples were observed at the following condition: 15 kV, 0.055 Pa, and magnification of 900×.

### 2.3. Pasting Properties

HAAKE RheoStress 1 (Thermo Fisher Scientific Brno s.r.o., Brno, Czech Republic) was used for assessing the pasting properties of flours. The suspension was prepared by mixing (6.0 ± 0.1) g of the flour with (30.0 ± 0.1) g of water. The rotation temperature ramp was performed using coaxial cylinders Z34 DIN Ti with a gap of 7.2 mm. The viscosity of a sample was measured over a given period of time while it was stirred. The test was divided into five stages: the addition of water to the flour sample; the heating phase; holding at maximum temperature; the cooling phase, and a final holding stage. The profile was: the addition of water to the flour sample, holding at the temperature of 30 °C for 120 s, heating to 90 °C for 220 s, holding at 90 °C for 300 s, cooling to 40 °C for 220 s and holding at 40 °C for 120 s. The suspension was stirred at 160 rpm during the test [25]. Each test was performed on samples prepared with at least three replicates. The results are represented as mean values.

### 2.4. Dough Formula

The basic dough formula consisted of flour (100 g), water (90 g), sucrose (1.86 g), salt (1.50 g) and dry yeast (1.80 g). The amounts of all ingredients were related to flour dry matter.

### 2.5. Uniaxial Elongation Test

Samples used for the uniaxial deformation test were prepared according to a dough formula without yeast. The dough was shaped into thin rolls, put onto the lubricated surface of a Teflon mold, and compressed with a lubricated top plate. Test pieces of dough were formed into 5 cm long pieces with a trapezoidal cross-section (3 mm, 5 mm, 4 mm). The doughs were kept for (40 ± 2) min at (30 ± 2) °C. Uniaxial elongation tests were performed using a texture analyzer TA.XT plus (Stable Micro Systems Ltd., Godalming, United Kingdom) equipped with an SMS/Kieffer Dough and Gluten Extensibility Rig. The measuring conditions were as follows: measure force in tension, pre-test speed 2.00 mm/s, test speed 3.00 mm/s, post-test speed 10.00 mm/s, and trigger force 5 g. The force required to stretch the dough sample and the displacement of the hook were recorded as functions of time. The values of importance were the peak force *R* (mN), i.e., resistance to extension, and the distance at which this peak force occurs, which is expressed as dough elongation *E* (mm). Extension area *A* (mN/mm) is the area under the curve, which is proportional to the energy required to stretch the test piece until it ruptured. At least six measurements were performed. The results are represented as mean values.

### 2.6. Bread Baking

Dry yeast was reactivated for (10 ± 1) min in a sugar solution (35 ± 1) °C prepared from sucrose and 60 mL of water. The flour, sugar solution, salt, and the rest of the water were placed into an Eta Gratus mixer bowl (ETA a.s., Prague, Czech Republic) and mixed for (6 ± 1) min. The prepared dough (600 g) was scaled into bread pans of 9.4 × 18.3 × 7.0 cm and placed into a proofer for (20 ± 2) min at (30 ± 1) °C and 85% relative air humidity. The loaves were baked for (40 ± 2) min at (180 ± 5) °C (MIWE cube, Pekass s.r.o., Plzeň, Czech Republic). The baked bread loaves were removed from the pans and stored at room temperature (21 ± 3) °C for 2 h. Loaf volume was determined using plastic granulates the size of rape seed. Loaf-specific volume (mL/g) was obtained by dividing the bread volume by bread weight. Three batches of three varieties of bread were baked for each flour. The results are represented as mean values.

Texture profile analysis (TPA) was performed on TA.XT plus texture analyzer (Stable Micro Systems Ltd., UK) was used to test the textural properties of the breadcrumb. The test was carried out on bread samples 35 mm in diameter and 10 mm in height obtained from the center of each loaf. Each sample was placed on the analyzer base and squeezed twice with a 75.0 mm diameter cylinder probe P/75. Parameters of the test were: pre-test speed 1.00 mm/s; test speed 5.00 mm/s, strain 40%, and trigger force 5 g. Crumb parameters (hardness, springiness, cohesiveness, resilience, and chewiness) were then determined [26]. At least three samples obtained from each bread were tested. The results are represented as mean values.

### 2.7. Statistical Analysis

The results were statistically analyzed using analysis of variance (ANOVA). The differences between samples were tested on a 0.05 significance level using the Fisher LSD test. Principal component analysis (PCA) was used to illustrate the relationship between variables and samples. All statistical analyses were performed using Statistica 13.0 (TIBCO Software s.r.o., Prague, Czech Republic).

## 3. Results and Discussion

### 3.1. Flour Characteristics

The tested flours significantly varied in their contents of starch, proteins, fibers, sugars, and the Hagberg falling number (Table 1). These results were expected since the grain characteristics are known to be influenced by the type of rice, growing conditions, postharvest operations, and other factors [17,27]. Black, red, and glutinous flours exhibited significantly higher protein and fiber contents, as well as lower starch content than refined white flours. The recorded values were in general agreement with previously published results [16,28]. High protein and fiber contents, together with lower starch content, might be the nutritional benefits of these flours in gluten-free bread baking.

**Figure 1:**
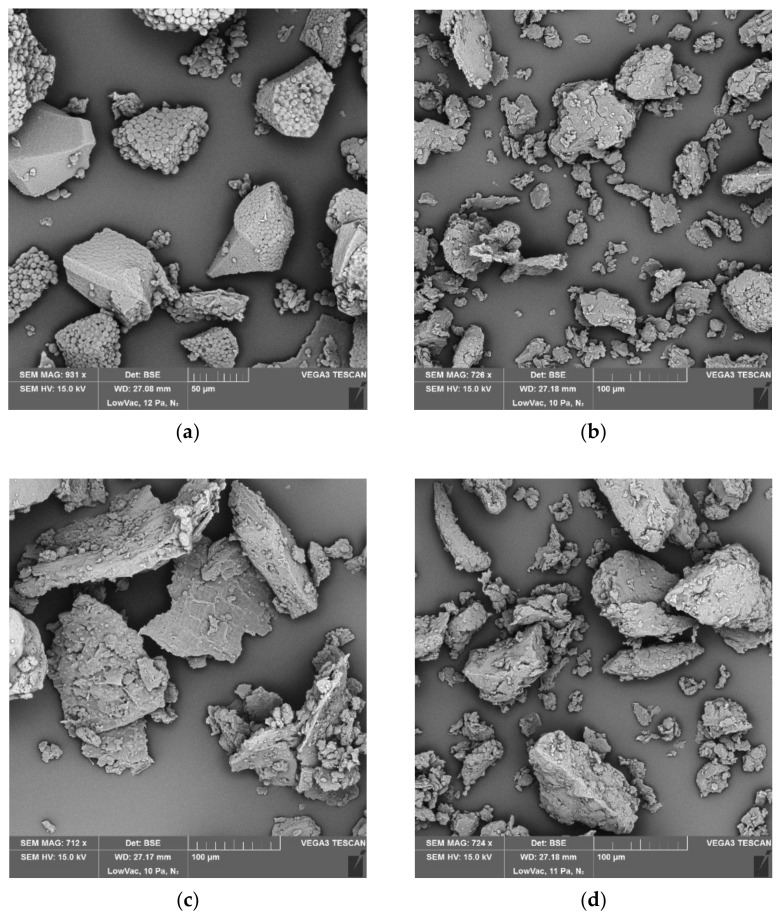

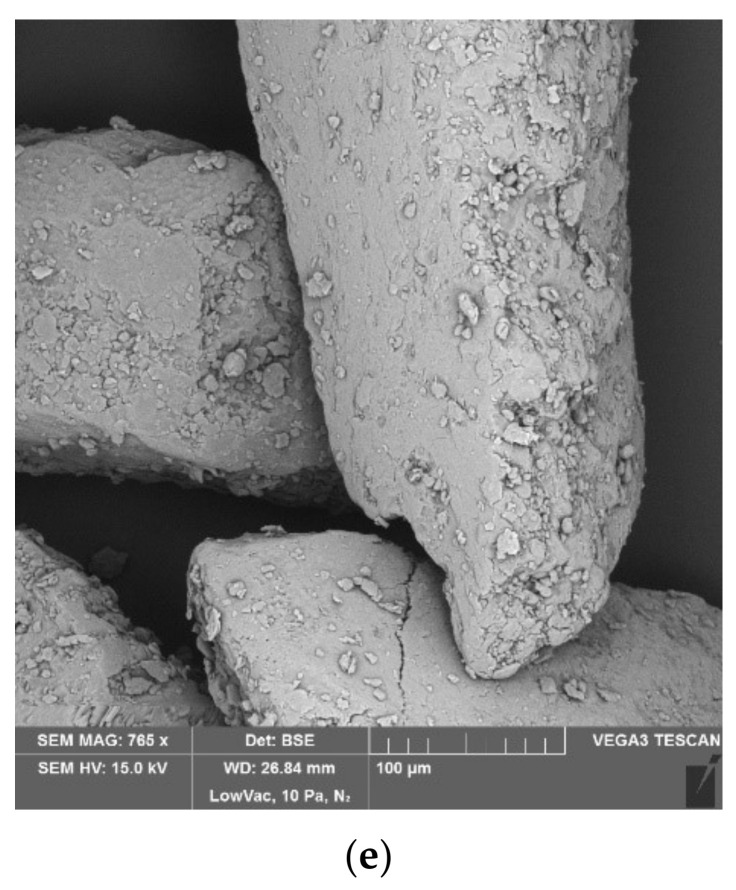
SEM of rice flours. (**a**) Black rice: compact polyhedral endosperm cells (50–100 µm diameter) showing visible individual starch granules in the protein matrix. A few pieces of small clusters (10–30 µm diameter) of starch granules and protein matrix; (**b**) Glutinous rice: large (50–80 µm diameter) and small (<30 µm diameter) endosperm cells of irregular shapes showing starch granules embedded in a protein matrix. Fragments of protein (<10 µm diameter); (**c**) Red rice: endosperm cells (100–200 µm) showing starch granules embedded tightly in the protein matrix. Individual starch granules are not visible. A few pieces of protein fragments (<15 µm); (**d**) White rice—refined fine flour: large (70–100 µm diameter) and small (<30 µm diameter) endosperm cells of irregular shapes showing starch granules embedded in the protein matrix. Fragments of protein (<5 µm diameter); (**e**) White rice—refined semi-coarse flour: Large blocks of endosperm (400–600 µm).

The content of damaged starch was observed in the range of (2.0–10.7)%. The flours from polished japonica rice with 4% and 6% content of damaged starch were previously found to have good characteristics and be suitable for gluten-free bread making [29]. Even if white semi-coarse flour, red flour, and glutinous flour can fit this range, it is not clear whether the conclusions [29] are easily applicable to the other types of rice. The variations in the amount of damaged starch granules were probably impacted by the differences in grain composition, as well as grain hardness [28], which determined the grain response to mechanical forces applied during grinding. The different grain response to mechanical forces is also evident from various shapes of flour particles (Figure 1). The content of damaged starch was, moreover, related to flour granulation since it significantly increased with decreasing flour particle size.

### 3.2. Dough Behavior during Uniaxial Elongation Test

A relatively wide range of resistance to extension *R* (92–250) mN, extensibility *E* (2.8–7.6) mm and area under curve *A* (74–1020) mN·mm were recorded in the tested doughs (Table 2). The doughs prepared from pigmented and glutinous rice exhibited generally weaker behavior, and their response to elongation was probably influenced by the presence of bran particles. The presence of these parts of grain in the flour is necessary since anthocyanin pigments are situated in the grain coat, and bran particles increase the fiber content in bread as well. A similar impact of bran particles was reported by Packkia-Doss et al. [9]. Additionally, the reduced resistance to extension recorded in the doughs made from glutinous and red rice may be related to a higher content of damaged starch in these flours. This similar reduction was previously reported on wheat dough prepared from flour with a high content of mechanically modified starch [30]. Besides the presence of bran particles, the content of damaged starch was another factor influencing dough behavior. Finally, the differences in the chemical composition of starch granules and the content of amylose and amylopectin reported by da Silva et al. [31] could also play a role in dough behavior.

The behavior of doughs prepared from the refined white flours was influenced mainly by the size of flour particles. Large-sized particles strengthened dough from semi-coarse flour. This was recorded as an increase in resistance to extension and the energy required for dough elongation. The differences between doughs prepared from two refined white flours were more evident than the differences in behavior among doughs from glutinous and red rice. It may indicate that the dough response to uniaxial elongation was influenced by flour granulation rather than other factors (rice type, presence of bran particles, or flour composition). However, further research is necessary to confirm this hypothesis. The relation between dough behavior during the uniaxial elongation test and bread quality is well described in the wheat dough. However, it is not clear whether uniaxial elongation is a suitable tool for the prediction of gluten-free bread quality.

### 3.3. Pasting Properties

The viscosity of the slurries at 30 °C varied at the range of (2.7–8.9) mPa (Table 3). The slurries with glutinous and red rice flours exhibited lower viscosity *η*_30°C_ than the slurries with refined flours. The viscosity of the slurries with all types of fine flours was, moreover, lower than the slurry with semi-coarse flour. The slurry with black rice was the only exception since its viscosity was the highest among the tested flours. The slurries prepared with glutinous and red rice flours exhibited a shift to a lower pasting temperature *T*_0_, lower values of *η_Peak_* and *η_Final_* as well as higher values of breakdown and setback region compared to the slurries with refined flours. The decrease in the effective concentration of starch, together with competition for water between bran particles and starch, may explain the lower viscosity in the slurries with red and glutinous flours [32,33]. High water retention of bran particles probably impacted the redistribution of water among substances and increased the values of setback region and total setback.

The slurry prepared from black rice flour exhibited the highest values of viscosity *η*_0_ and breakdown, together with the lowest values of *η_Final_*, setback region and total setback. The slurry viscosity during the initial and heating stages was probably increased by the high-water binding and holding capacity of the substances present in this flour. A rapid decrease of viscosity during the holding and cooling stages may be explained by the extensive release of water from previously hydrated substances. It may be hypothesized that this behavior was closely related to proteins. The proteins probably bound an extensive amount of water, which was subsequently released from the denatured proteins. Starch and fibers were not able to absorb such an extensive amount of water, leading to a decrease in viscosity due to the presence of an extensive amount of free water. Low values of setback region and total setback indicated low tendency to retrograde [34]. The presence of free water released from proteins and its dilution effect may also explain this observation.

### 3.4. Bread Characteristics

**Figure 2:**
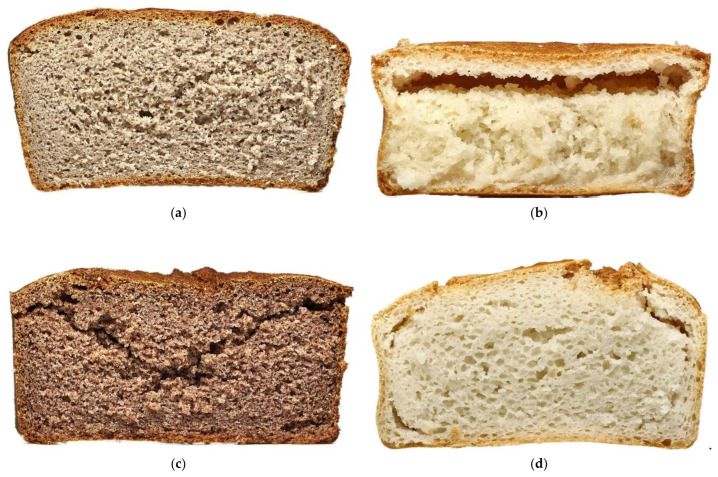

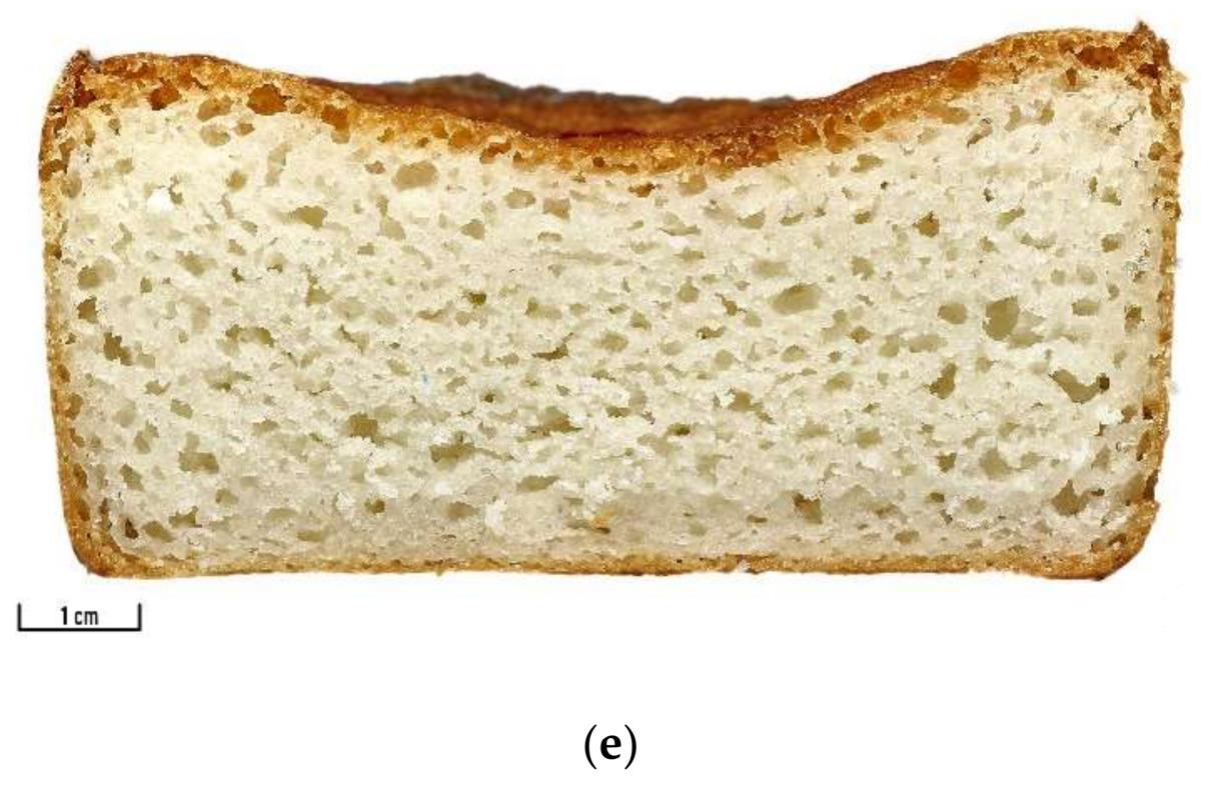
Crumb of bread prepared from (**a**) black rice; (**b**) glutinous rice; (**c**) red rice; (**d**) refined white fine; (**e**) refined white semi-coarse flour.

The various loaves of bread prepared from different refined white flours had relatively large pores (Figure 2d,e), but the bread had low volumes and hard crumbs (Table 4). These characteristics may be related to the thick layer of dough surrounding the pores. The high adhesiveness of these loaves of bread may be explained by the high amount of water available for starch gelatinization and retrogradation during bread baking and cooling. A part of water probably remained free in these doughs, while it was absorbed by bran particles in black, red, and glutinous flours.

The negative impact of bran particles on bread volume and bread hardness [8,9] was not observed. Moreover, the loaves of bread prepared from black, red, and glutinous rice flours exhibited higher loaf-specific volume and springiness, as well as lower hardness, cohesiveness, resilience, and chewiness than the loaves of bread from refined flours (Table 4). The significance of the differences varied among the parameters. The increase in loaf volume was significant only in bread from red flour. Some defects decreased the quality of loaves of bread prepared from the red and glutinous rice flours. A crack from the center to the upper corners (Figure 2c) decreased the integrity of the red bread crumb, which probably influenced the values of crumb hardness, cohesiveness, and chewiness. The value of the loaf-specific volume of bread from glutinous flour was overrated by the presence of one large pore situated under the crust (Figure 2b). This part of the crust collapsed during cutting, flattening the loaf. The insufficient thickness of the continuous glutinous bread crumb made taking a sample of the required size difficult, which may misrepresent the values of textural characteristics, mainly the hardness and chewiness. The ability of the crumb to regain its original height (resilience) was weak in this bread due to high crumb stickiness, which is evident from the high value of adhesiveness. High adhesiveness may be related to the high content of amylopectin in starch [15].

The crumb of the bread prepared from black flour did not exhibit any defect (Figure 2a). The crumb hardness and chewiness were higher than in the loaves of bread from glutinous and red flours, but the values of bread from refined flours were not reached.

Even if the study was mainly focused on testing flour, dough, slurry, and bread characteristics, together with relations among them, the informal sensory evaluation was also performed. Bread prepared from glutinous flour had sticky crumbs, with a neutral taste and flavor. Similar neutral tastes and flavors were recorded in loaves of bread from refined white flours. The evaluation of breads from pigmented flours varied among the evaluators. Some of them found the color of these loaves of bread attractive, while others found them unacceptable. The evaluators recognized intensive flavor and taste in these loaves of bread. The flavor of bread from black rice flour was described as fruity and floral, while the flavor of bread from red rice flour was leafy, floral, nutty or amaranth-like. The recorded intensive flavor and taste may impact the applicability of these flours in bread making. The blends of black and red rice flours with white rice or glutinous flours may decrease the intensity of flavor and taste to an acceptable level. A formal sensory evaluation analysis has to be performed to confirm these assumptions.

### 3.5. The Relations among Flour, Slurry, Dough, and Bread Parameters

**Figure 3:**
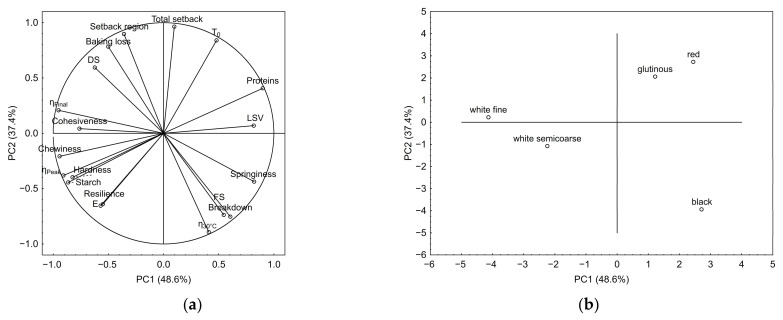
Principal component analysis: (**a**) score plot for the first and second principal components; (**b**) loading plot for the first and second principal components.

Two principal components (PC) were located to describe 86.0% of the total data variance. The principal component loadings (Figure 3a) showed that the loaf-specific volume, cohesiveness, final viscosity, and chewiness strongly influenced PC1 (48.6%), while the second principal component (37.4%) is mainly formed by total setback and setback region.

Bread characteristics (cohesiveness, chewiness, hardness, and resilience) were closely related to each other and strongly positively correlated with the content of starch in flour, slurry peak viscosity *η_Peak_*, as well as final viscosity *η_Final_*. It is evident that starch content significantly influenced bread characteristics since starch is a major component of the flours used. Crumb hardness was related to slurry viscosity and its increase during gelatinization. Peak viscosity *η_Peak_* had a stronger impact on the crumb hardness than the final viscosity *η_Final_*. This observation was evident mainly from the results of black rice flour. The peak viscosity of slurry with this flour was higher than in slurries with glutinous and red rice flours (black rice: 210 mPa·s; glutinous rice: 113 mPa·s; red rice: 119 mPa·s), final viscosity was lower (black rice: 130 mPa·s; glutinous rice: 252 mPa·s; red rice: 280 mPa·s) and hardness was higher (black rice: 12.7 N; glutinous rice: 5.1 N; red rice: 8.8 N). As can be seen in Table 3, the slurry viscosity and its increase during the heating stage of the test varied among tested flours, confirming the strong impact of rice type.

Crumb hardness and resilience were positively correlated with dough extensibility E during the uniaxial deformation test. The correlation of bread characteristics with the other characteristics recorded during the uniaxial elongation test (dough resistance to extension, area under curve) was very weak. Even if dough behavior during this test is often used to predict the bread-baking performance of wheat flour, this test seems to be of minor importance in gluten-free bread baking.

Breadcrumb cohesiveness, chewiness, hardness, and resilience were negatively correlated with protein content. Even if proteins present in gluten-free flour are known to exhibit quite different behavior compared to wheat gluten, they are probably able to decrease crumb hardness. This effect is desirable, since hard bread crumb is typical in gluten-free bread.

Bread springiness and baking loss were negatively correlated. The baking loss was positively correlated with the content of damaged starch and setback region and negatively with breakdown, the content of maltose (sugars), and viscosity at the initial stage of the test. The processes taking place in the slurry during the test may differ from the processes occurring in the dough during baking since the slurry is prepared using a significantly higher amount of water than is present in the dough during baking. However, it is evident that baking loss was strongly influenced by the content of free water in the dough during baking and cooling. If the dough or bread contained free water, this water evaporated during bread baking and cooling, increasing baking loss. Water bound in starch, proteins and brans was kept in the dough and bread, decreasing baking loss. The baking loss also rose with an increasing amount of damaged starch. The damaged starch granules are known to have higher water absorption ability, but this water is released during heating [35] and evaporated during baking. Qin et al. [29] found the content of damaged starch in the range of 4–6% to be the most suitable for bread. It is not possible to simply confirm their observation since generally better characteristics were recorded in bread from black rice flour with 2% of damaged starch. A possible explanation is that the optimal content of damaged starch depends on the rice type.

As can be seen from the scatterplot (Figure 3b), the characteristics of doughs and loaves of bread prepared from black, red, and glutinous rice flours exhibited different characteristics than those recorded in white rice dough and bread. The differences were recorded mainly in final slurry viscosity during the test, in the loaf-specific volume and bread crumb cohesiveness. Flours from glutinous and red rice were characterized mainly by the total setback and temperature *T*_0_. Black rice flour was characterized mainly by slurry viscosity at the beginning of the test (*η*_0_) and breakdown. The impact of flour granulation on slurry and bread characteristics was also recorded in the samples prepared from white rice.

## 4. Conclusions

The tested flours prepared from pigmented (black and red), glutinous, and common rice exhibited different characteristics. The type of rice also influenced flour pasting characteristics and dough behavior during the uniaxial deformation test. The characteristics of loaves of bread were mainly impacted by flour pasting properties. The loaves of bread prepared from black, red, and glutinous rice flours exhibited higher loaf-specific volume and springiness, as well as lower hardness, cohesiveness, resilience, and chewiness than loaves of bread from refined flours. The significance of the differences varied among the parameters. The bread prepared from black rice flour was the only one without any crumb defects. A crack from the center to the upper corners was observed in bread from red rice flour. One large pore was situated under the crust of the bread from glutinous rice. Moreover, the crumb of this bread was sticky. The tested whole-meal flour prepared from glutinous rice exhibited a good baking performance and has the potential to become a nutritionally valuable ingredient in gluten-free baking. The informal sensory evaluation revealed the intensive flavor and taste in loaves of bread prepared from pigmented rice flours, which may limit their applicability in gluten-free bread baking. The intensity of flavor and taste may be expected to be decreased by the blending of these flours with other gluten-free flours of neutral flavor and aroma. The breadmaking potential of these blends should be the goal of the next research.

## Figures and Tables

**Table 1:** Content of starch, proteins, fibers, sugars, and damaged starch (DS) in tested flours in g/100 g, Hagberg falling number (HFN) in s ^1^.

Flour Type	Starch	Proteins	Fibers	Maltose	DS	HFN
Black	76.6 ± 0.9 b	8.9 ± 0.9 b	2.5 ± 0.4 b	0.88 ± 0.02 a	2.0 ± 0.2 e	256 ± 30 c
Glutinous	76.2 ± 0.8 b	9.7 ± 0.5 a	2.8 ± 0.3 b	0.07 ± 0.02 d	6.7 ± 0.2 b	87 ± 9 d
Red	74.0 ± 0.9 c	10.0 ± 0.8 a	7.0 ± 0.4 a	0.34 ± 0.03 b	6.1 ± 0.2 c	592 ± 20 a
White fine	79.0 ± 0.3 a	7.0 ± 0.5 c	0.44 ± 0.09 c	0.19 ± 0.02 c	10.7 ± 0.8 a	447 ± 20 b
White semi-coarse	79.0 ± 0.5 a	7.0 ± 0.9 c	0.40 ± 0.07 c	0.17 ± 0.04 c	4.0 ±0.5 d	466 ± 20 b

^1^ The mean values ± standard deviation followed by different letters in the column differ significantly (*p* < 0.05).

**Table 2:** Behavior of doughs during the uniaxial elongation test (dough resistance to extension *R*, dough extensibility *E*, and area under curve Area) ^2^.

Flour Type	*R* (mN)	*E* (mm)	Area (mN·mm)
Black	180 ± 10 b	6.5 ± 0.5 b	608 ± 20 c
Glutinous	92 ± 5 c	2.3 ± 0.2 c	74 ± 10 d
Red	84 ± 6 c	2.8 ± 0.5 c	87 ± 10 d
White fine	190 ± 20 b	7.6 ± 0.6 a	692 ± 20 b
White semi-coarse	250 ± 8 a	7.6 ± 0.8 ab	1020 ± 30 a

^2^ The mean values ± standard deviation followed by different letters in the column differ significantly (*p* < 0.05).

**Table 3:** Pasting properties of flours (*η*_30°C_ viscosity at 30 °C, *T*_0_ temperature when viscosity started to rise, *η_Peak_* peak viscosity, breakdown, *η_Final_* final viscosity, setback region, total setback) ^3^.

Flour Type	*η_30°C_* mPa·s	*T_0_* °C	*η_Peak_* mPa·s	Breakdown mPa·s	*η_Final_* mPa·s	Setback Region mPa·s	Total SetbackmPa·s
Black	8.94 ± 0.14 a	51 ± 1 c	210 ± 10 c	159 ± 10 a	130 ± 10 c	–80 ± 9 d	79 ± 3 d
Glutinous	2.70 ± 0.10 a	60 ± 2 b	113 ± 10 d	22 ± 3 b	252 ± 10 bc	139 ± 9 b	160 ± 10 b
Red	2.89 ± 0.21 cd	65 ± 2 a	119 ± 16 d	27 ± 4 b	280 ± 9 c	160 ±7 a	188 ± 5 a
White fine	3.15 ± 0.07 c	66 ± 2 a	416 ± 17 a	7 ± 2 c	537 ± 9 a	120 ± 9 c	127 ± 2 d
White semi-coarse	3.67 ± 0.06 b	67 ± 2 a	359 ± 20 b	10 ± 5 c	481 ± 4 a	122 ±7 c	132 ±9 d

^3^ The mean values ± standard deviation followed by different letters in the column differ significantly (*p* < 0.05).

**Table 4:** Textural characteristics of breads prepared from different rice flours ^4^.

Flour Type	Loaf Specific Volume (mL/g)	Baking Loss (%)	Hardness (N)	Springiness (%)	Cohesiveness (%)	Resilience (%)	Chewiness (-)	Adhesiveness (mN·s)
Black	1.7 ± 0.4 ab	14 ± 3 c	12.7 ± 0.9 c	82 ± 2 a	68 ± 9 b	45 ± 2 ab	67 ± 9 c	22 ± 9 d
Glutinous	1.3 ± 0.4 ab	20 ± 1 ab	5.1 ± 0.7 e	75 ± 2 b	75 ± 3 b	34 ± 4 c	29 ± 4 e	626 ± 100 a
Red	1.9 ± 0.3 a	22 ± 1 ab	8.8 ± 0.6 d	75 ± 2 b	63 ± 9 b	40 ± 3 bc	41 ± 6 d	41 ± 20 d
White fine	1.2 ± 0.4 b	24 ± 3 a	22.9 ± 0.3 b	64 ± 9 c	89 ± 9 a	47 ± 3 a	250 ± 10 a	189 ± 100 b
White semi-coarse	1.1 ± 0.3 b	18 ± 2 bc	26.6 ± 0.9 a	75 ± 9 ab	69 ± 7 b	46 ± 2 a	160 ± 20 b	93 ± 10 c

^4^ The mean values ± standard deviation followed by different letters in the column differ significantly (*p* < 0.05).

## Data Availability

Results are available from the corresponding author.
